# A modified 384‐well‐device for versatile use in 3D cancer cell (co‐)cultivation and screening for investigations of tumor biology in vitro

**DOI:** 10.1002/elsc.201700008

**Published:** 2017-11-24

**Authors:** Miriam Widder, Karen Lemke, Bünyamin Kekeç, Tobias Förster, Andreas Grodrian, Gunter Gastrock

**Affiliations:** ^1^ Department of Bioprocess Engineering Institute for Bioprocessing and Analytical Measurement Techniques e.V. Rosenhof Heilbad Heiligenstadt Germany

**Keywords:** 3D cell culture, Automation, Pancreatic cancer, Substance screening, Tumor‐stroma‐interaction

## Abstract

Pancreatic cancer exhibits a worst prognosis owed to an aggressive tumor progression i.a. driven by chemoresistance or tumor‐stroma‐interactions. The identification of candidate genes, which promote this progression, can lead to new therapeutic targets and might improve patient's outcome. The identification of these candidates in a plethora of genes requires suitable screening protocols. The aim of the present study was to establish a universally usable device which ensures versatile cultivation, screening and handling protocols of cancer cells with the 3D spheroid model, an approved model to study tumor biology. By surface modification and alternative handling of a commercial 384‐well plate, a modified device enabling (i) 3D cultivation either by liquid overlay or by a modified hanging drop method for (ii) screening of substances as well as for tumor‐stroma‐interactions (iii) either with manual or automated handling was established. The here presented preliminary results of cell line dependent dose‐response‐relations and a stromal‐induced spheroid‐formation of the pancreatic cancer cells demonstrate the proof‐of‐principle of the versatile functionality of this device. By adapting the protocols to automation, a higher reproducibility and the ability for high‐throughput analyses were ensured.

**Abbreviations**
2/3D: two/three dimensional
HPSC: human pancreatic stellate cells
LO: liquid overlay
MHD: modified hanging drop
PDAC: pancreatic ductal adenocarcinoma
pHEMA: poly(2‐hydroxyethyl methacrylate)
w/wo: with/without
wt: wildtype

## 1 Introduction

Pancreatic cancer is one of the leading causes of cancer related deaths worldwide 1. Among others this is owed to limited therapeutic options caused by a high degree of resistances to conventional chemotherapeutics and an aggressive tumor progression 2, 3, 4.

Chemoresistance can occur due to intrinsic (de novo) or acquired (during therapy) characteristics of cancer cells 4 including mechanisms like an enhanced drug efflux or an alteration of the drug targets 5. Another crucial role for tumor progression is awarded to interactions of the malignant cancer cells with their surrounding non‐malignant connective tissue, the stroma. Main constituents of the stroma of pancreatic cancer are immune cells, extracellular matrix and pancreatic stellate cells 6. The direct 7 and/or indirect 8 cross‐talk between the malignant and non‐malignant parts finally leads to an activated stroma which favors tumor progression through several mechanisms 9. Considering that the stroma can take an amount of over 90% of the total tumor mass 10, tumor‐stroma‐interactions might play a prominent role in pancreatic cancer. In order to overcome resistances, new therapeutic targets, e.g. tumor‐stroma‐interactions or totally new drugs have to be identified 4, 11, 12.

The discovery of such targets is associated with a huge effort because just the minority of putative candidates acts as a potential hit and can be used for further anticancer drug development. Thanks to automation, thousands of putative targets can nowadays be analyzed by high‐throughput screenings in vitro 13. Nevertheless, most of the promising candidates fail in further clinical testing 14 which can be attributed to the artificial character of the performed 2D‐assays which barely reflect important features of solid tumors in vivo – cell‐cell and cell‐matrix‐interactions in three dimensions 15. A better in vitro model is the 3D spheroid, developed during cultivation of adherent cells under non‐adhesive conditions, e.g. in hanging drops 16 or on agarose coated surfaces (liquid overlay) 17. The presence of less artificial cell‐cell and cell‐matrix‐interactions within 3D cultures 14 leads to in vivo‐like growth kinetics 18, metabolic rates 19 and chemoresistances 20 and make achieved data more credible in respect of subsequent animal testing compared to 2D culture. However, implementation of 3D cultures to large‐scale studies is cost‐intensive and time‐consuming.

The aim of the present study was to establish a device with universal versatility for 3D cancer cell cultivation and screening to ensure a high degree in flexibility of choosing the perfect 3D (co‐)cultivation method and screening approach of any cell sample.

## 2 Materials and methods

### 2.1 Cell lines and 2D culture

The Pancreatic Ductal Adenocarcinoma (PDAC) cell lines PaTu‐8988t (wildtype and two doxycycline‐inducible mutants *clone I* and *clone II*) and Suit2‐007 (wildtype) as well as human pancreatic stellate cells (HPSC) were kindly provided by PD Dr. Malte Buchholz (Philipps University, Center for Tumor Biology and Immunology, Marburg, Germany). The human colorectal carcinoma cell line HT29 was obtained from the DSMZ (ACC‐299). PDAC cell lines were obtained in DMEM (high glucose, Sigma Aldrich, München, Germany) with 10% v/v FBS (Biochrom, Berlin, Germany), 100 U/mL penicillin/100 μg/mL streptomycin (Biochrom) and 2 mM L‐glutamine (Sigma Aldrich). HPSC were cultivated in DMEM/F12 (Gibco, Thermo Fisher Scientific, Schwerte, Germany) with 10% v/v FBS and 100 U/mL penicillin/100 μg/mL streptomycin. HT29 were cultivated in RPMI (Sigma Aldrich) with 10% v/v FBS and 100 U/mL penicillin/100 μg/mL streptomycin. The cells were maintained as 2D cultures under standard conditions (95% relative humidity, 5% CO_2_, 37°C). Single cell suspensions were generated by trypsinization (trypsin‐EDTA 0.25%/0.02%; Sigma Aldrich). Seventy two hours prior to an experiment, cells were supplemented with or without (w/wo) 2 μg/mL doxycycline (Sigma Aldrich).

### 2.2 Modified 384‐well‐device for different 3D cell cultivation methods

384‐well plates (low volume, μClear‐bottom, Greiner Bio‐One, Frickenhausen, Germany) were adapted to different 3D cultivation techniques. The plates were prepared for liquid overlay method (LO) by coating each well with 10 μL agarose (0.7% w/v Seakem GTG agarose in PBS, Lonza, Basel, Switzerland). Plates were air dried for 10 min before the cells were seeded in 10 μL per well or alternatively sealed with parafilm and stored at 4°C. As a second technique, a new modified hanging drop method (MHD) was developed by overfilling the wells with 35 μL cell suspension and incubating the plates upside down in a humidified glass petri dish with spacers to avoid contact with the glass bottom. Prior to use, wells were coated as followed: pHEMA (Sigma Aldrich) was dissolved in 96% v/v ethanol in a final concentration of 5 mg/mL and filtered through a 0.2 μm CA‐membrane (Minisart, Sigma Aldrich). Wells were filled two times with 25 μL pHEMA per well in a tissue culture hood and left there until ethanol was evaporated. Plates were stored at room temperature until use.

### 2.3 Manual protocol for substance screenings in the modified 384‐well‐device

The substance screening should fit the following parameter: 72 h treatment of 3D cultures 24 h after cell seeding. The exponential proliferation phase of untreated cells should be ensured for 96 h. Therefor growth kinetics were preliminary performed with the LO‐method. Due to its lower volume, substrate limitations occur more likely in LO compared to MHD‐method. 100, 300, 500, 800, and 1000 cells per well were seeded with the respective medium supplemented w/wo doxycycline in a 4‐fold dissemination. On day 4 after seeding, cultures were fed with fresh medium, in case of LO by addition of 10 μL and in case of MHD by adding 17.5 μL after 17.5 μL were aspired. CellTiterGlo^®^3D assay was performed on day 2, 4, 6, and 8 after seeding as specified by the manufacturer and luminescence was measured on the Synergy H1 Multidetection Reader (BioTek, Bad Friedrichshall, Germany). Relative luminescence counts of each cell density were plotted against time in culture.

For substance screenings, cells were seeded in the determined density and the respective medium w/wo doxycycline with the LO‐ or MHD‐method. After cultivation under standard conditions for 24 h, gemcitabine or paclitaxel (Sigma Aldrich) was dissolved with the respective medium w/wo doxycycline. Dilutions were submitted to the cells in increasing concentration (0–100 μM). In case of LO‐method, 10 μL of the substance was added to each well and plates were cultivated under standard conditions. In case of MHD‐method, plates were incubated the right way up for 10 min to allow cell sedimentation. 17.5 μL medium was aspirated per well and 17.5 μL of the substance was added. Plates were incubated upside down under standard conditions. CellTiterGlo^®^3D assay was performed 72 h after treatment as mentioned above with the following modification: prior to the addition of assay reagent, medium was aspirated, in case of LO‐method 10 μL and in case of MHD‐method 25 μL. Relative luminescence units from untreated cells were adjusted to 100% and luminescence intensities from treated cells were scaled to it. IC_50_‐values were calculated on the basis of linear interpolation of adjacent values.

### 2.4 Manual protocol for a coculture assay in the modified 384‐well‐device

PDAC cells were mixed with increasing amounts of HPSC (0–100%) in 20% intervals in PDAC medium w/wo doxycycline. These mixtures were seeded at a density of 1000 cells per well either with LO‐ or MHD‐method. Plates were cultivated under standard conditions and monitored microscopically and photo documented 48 h after seeding (inverted microscope IX81 and software Cell^P, Olympus Deutschland GmbH, Hamburg, Germany).

### 2.5 Adaption of protocols to fully automated handling

#### 2.5.1 Pipet robot and software

Automated handling was performed with the Genesis 100/8 robot and the software Genesis 4.2 (Tecan, Männedorf, Switzerland). The robot housing (Fornax, Neustadt/Wied, Germany) was completely closed and guaranteed for antiseptic conditions through disinfection by 1.5% v/v lysoformine (Dr. Hans Rosemann GmbH, Berlin, Germany) and UV irradiation. All experiments were performed with conductive, pre‐sterilized tips (Fornax, Neustadt/Wied, Germany).

#### 2.5.2 Adaption of substance screening and coculture assay to automated handling

The modification of the 384‐well plates was adapted to automation. Coating with agarose or pHEMA was performed according to the parameters in 2.2 in part with homemade heating elements to warm up the agarose and the tips during the coating process. Except for preparation of cell suspension and gemcitabine dilutions, all steps of the protocol mentioned in 2.3 and 2.4., except for the invertation and cultivation of the plates, were adapted to automation with suitable devices under use of the liquid detection level of the robot.

## 3 Results and discussion

### 3.1 Experimental procedure and valuation of the modified 384‐well‐device

**Figure 1:**
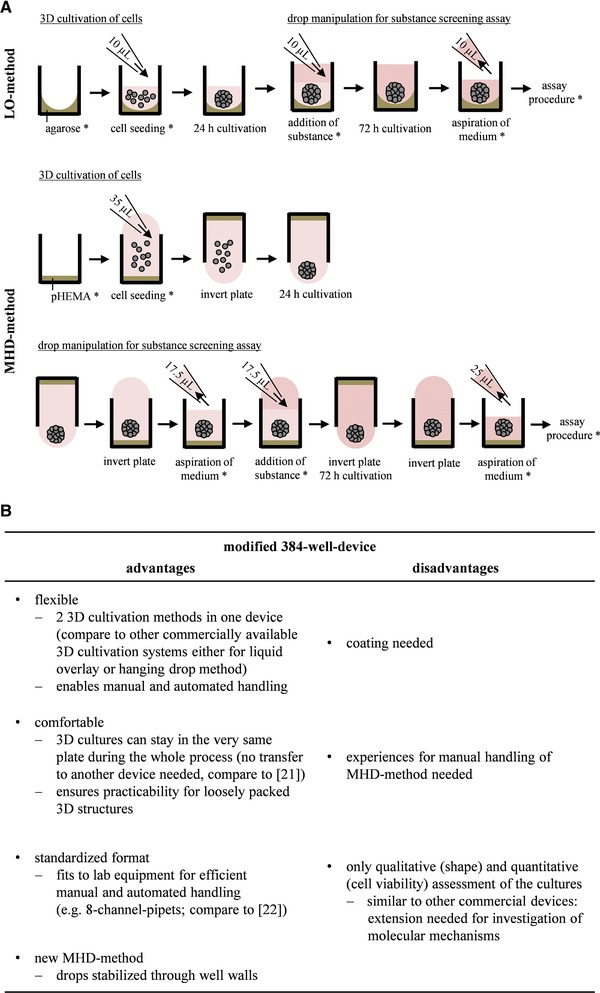
Schematic drawing of experimental procedure for LO‐ and MHD‐method within the modified 384‐well‐device. Here, steps depicted with * were automatically performed (A). Valuation of the modified 384‐well‐device by specifying its advantages and disadvantages (B).

The modified 384‐well‐device affords the opportunity of different 3D cell cultivation methods performed either manually or automated. An explanation of the handling followed by a numeration of the main dis‐ and advantages of the device is given in Fig. 1.

### 3.2 Substance screening of cultures in the modified 384‐well device

#### 3.2.1 The modified 384‐well device fulfills all requirements for 3D cell cultivation

To determine the linear cell proliferation range during the substance screening process, growth kinetics without substance were previously performed within the modified 384‐well‐device. A density and cell line dependent growth behavior was observed (Supporting Information Figs. 1 and 2). This is congruent to the finding that the proliferation index of 3D cultures depends on the cell type 23. Furthermore, doxycycline had no effect on the proliferation of wildtype cells and PaTu‐8988t *clone II* but inhibited expansion of PaTu‐8988t *clone I*. The determination of a differential proliferation in various cell lines within the present study confirms the usability of the modified 384‐well‐device to identify proliferation influencing candidate genes. Another important information is that a density of 300–500 cells per well allows an exponential proliferation of every analyzed cell line at least for up to 96 h after seeding.

#### 3.2.2 The modified 384‐well‐device ensures manual and automated substance screenings within different 3D cultivation methods

**Figure 2:**
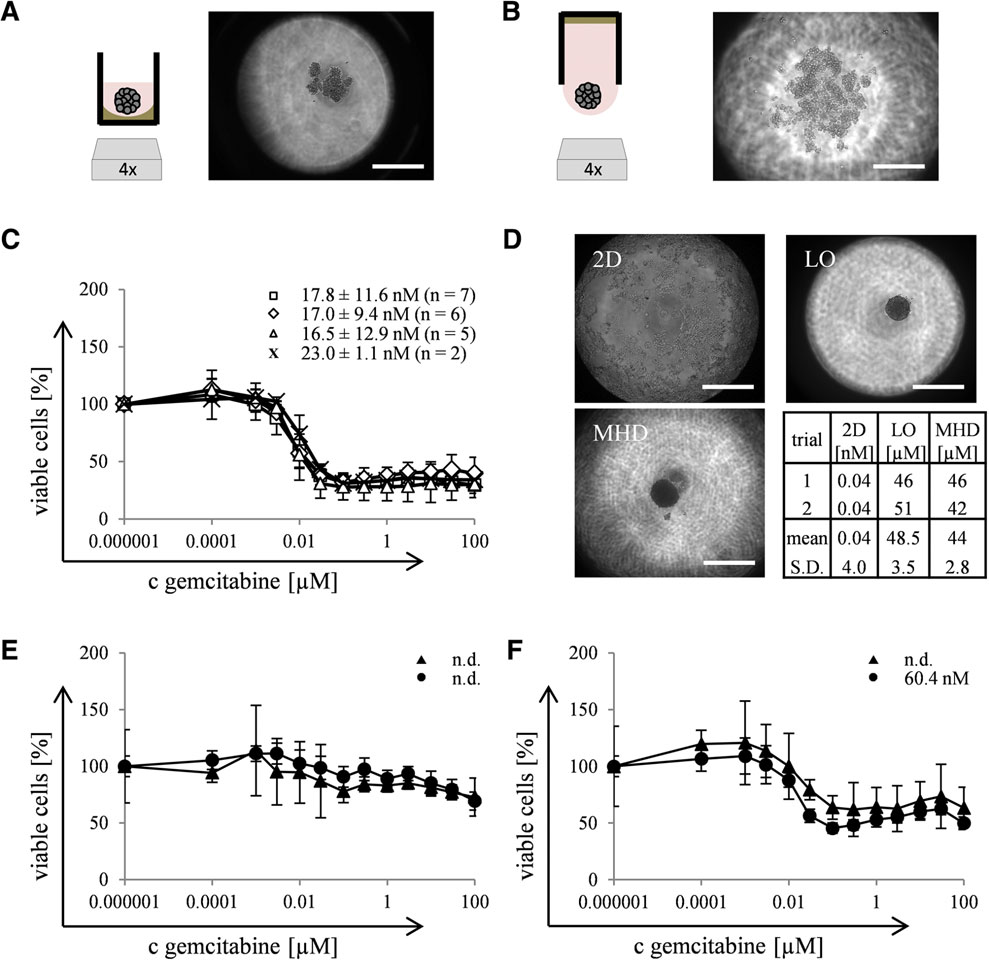
Suitability of the modified 384‐well‐device for substance screenings. Scheme and photo documentation of PaTu‐8988t wildtype cells cultivated 48 h with LO‐ (A) and MHD‐method (B). Dose‐response‐relations and calculated IC_50_‐values of PaTu‐8988t wildtype cells treated with gemcitabine. Several independent approaches (*n*) were performed manually with LO‐method (□, *n* = 7) or automatically with LO‐ (◇, *n* = 6) or MHD‐method (△, *n* = 5). Manually performed analyses with 2D cultures (×, *n* = 2) serve as control (C). Automated microscopic determination of HT29‐cells cultivated 2D or 3D with LO‐ and MHD‐method and calculated IC_50_‐values from these cultures treated with paclitaxel in two independent approaches (D). Dose‐response‐relation and calculated IC_50_‐values of two doxycycline‐inducible mutants from PaTu‐8988t: *clone I* (E) and *clone II* (F) treated with gemcitabine in two independent approaches (LO‐method automatically ▴ and manually •). Data are depicted as mean +/‐ SD with 12 samples contributing to each concentration of every individual approach n. Bar – 500 μm.

In a next step, operational capability of the modified 384‐well‐device for substance screenings was tested. Experiments with PaTu‐8988t wildtype cells (3D cell cultivation, microscopic determination, and substance screening) were performed manually before the protocols were implemented to automated handling. In the present study a slight tightening of the 3D cultures in LO‐ compared to MHD‐method was observed (Fig. 2A and B). Zanoni already reviewed, that different 3D cultivation techniques differentially influence the shape of the cultures 24, showing the necessity of being flexible in choosing the right cultivation technique for each cell sample.

By introducing the modified 384‐well‐device to the substance screening procedure, the MHD‐method was determined as unsuitable for manual handling due to the necessity of a defined immersion depth for pipetting. Instead, the LO‐method led to reproducible results when handled manually: Dose‐response‐relations were recorded with a mean IC_50_‐value of 17.8 nM (Fig. 2C). After the automation, reproducible and comparable IC_50_‐values could be determined with LO‐ (17.0 nM) as well as MHD‐method (16.5 nM), depicting that both methods are suitable for substance screenings. Furthermore, IC_50_‐values from automated handling were comparable to those from manually performed experiments with the LO‐method, depicting that automation did not influence the process.

Additionally, a comparison of the dose‐response‐relation reveals slight increased IC_50_‐values of 2D (23.0 nM) compared to 3D cultures (16.5–17.8 nM; Fig. 2C). This fact disagrees with the omnipresent opinion that 3D cultures are more resistant compared to 2D cultures 25. Among others, the limited diffusion of substances and hypoxia within tight spheroids are discussed to be involved in the drug resistance 26. The cultures in the present study are packed very loosely, even 8 days after seeding (Supporting Information Fig. 2), so the mentioned mechanisms for chemoresistance may be nonexistent. Aside from that, it is well accepted that 3D cultures can also show an increased sensitivity compared to 2D cultures, e.g. toward specific substances like kinase inhibitors 27. Anyway, to exclude any impracticalness of our modified 384‐well‐device and substance screening procedure, experiments were performed with HT29‐cells which form tight spheroids 24 h after seeding (Fig. 2D). Again, comparable IC_50_‐values could be observed for LO‐ (48.5 μM) and MHD‐method (44 μM). Furthermore these values show a huge difference to the IC_50_‐value from 2D culture (0.04 nM), confirming the suitability of the modified 384‐well‐device for substance screenings of 3D cultures. Finally, it leads to the conclusion that the shape of 3D cultures and their response to chemotherapy is cell‐line‐dependent and not influenced by the modified 384‐well‐device, contrary, it helps to deal even with loosely packed 3D cultures.

#### 3.2.3 The modified 384‐well‐device ensures substance screenings in various cell lines

In a last step the versatility of the modified 384‐well‐device was tested with independent doxycycline‐inducible PaTu‐8988t mutants. A differential influence of gemcitabine on *clone I* and *II* was detected (Fig. 2E and F). C*lone I* is impaired in proliferation through doxycycline and since substance screening was performed under submission of doxycycline, the detected resistance might be an artefact due to less expansion (Supporting Information Fig. 1). Those clones can be rated as dormant cells and might therefore not be useful for the screening of substances which target dividing cells 28. Convenient supplementary analyses of these clones can be performed with the assay presented by Wenzel et al. 29. It is also possible that the respective mutated genes are involved in the resistance mechanisms of both clones. The underlying mechanisms will be a topic for continuative investigations, e.g. by extension of the protocols to transcriptomic and metabolic profiling of selected clones.

To sum up, with the present proof‐of‐principle study, the usability of the modified 384‐well‐device for cell proliferation and substance screenings with different cultivation methods and operation modes was confirmed. The goal of the established assay was not to detect underlying resistance mechanisms but genes with an influence on proliferation and/or chemo‐response, as presented. These genes are the basis for further investigation of the molecular mechanisms of chemo‐resistance and can lead to the identification of new therapeutic options.

### 3.3 Coculture assay in the modified 384‐well‐device

#### 3.3.1 The modified 384‐well‐device is suitable for cocultivation of PDAC and stromal cells in different 3D cultivation methods and lead to the identification of stromal‐induced spheroid‐formation

**Figure 3:**
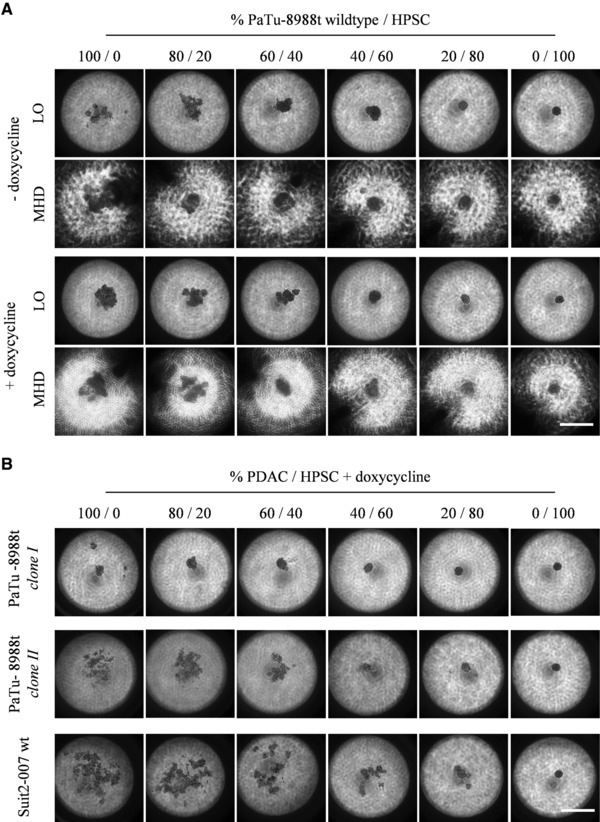
Investigation of tumor‐stroma‐interactions within the modified 384‐well device. Microscopic determination of PaTu‐8988t wildtype cells mixed with HPSC in different ratios seeded w/wo doxycycline either with LO‐ or MHD‐method and cultivated for 48 h (A). Microscopic determination of two doxycycline inducible mutants from PaTu‐8988t and Suit2‐007 wildtype cells mixed with HPSC in different ratios seeded with doxycycline in LO‐method and cultivated for 48 h (B). Bar – 500 μm.

To determine the usability of the modified 384‐well‐device for the cultivation and screening of cocultures, mixtures from PaTu‐8988t wildtype and HPSC were either cultivated with LO‐ or MHD‐method (Fig. 3A). A comparable HPSC‐induced spheroid‐formation of the PaTu‐8988t cultures in both cultivation methods was observed. Cultures rose in round shaped structure starting with 60% HPSC. Doxycycline had no influence on the shape of the cocultures. The stroma‐induced spheroid‐formation in PaTu‐8988t wildtype cells can be rated as tumor‐stroma‐interactions comparable to previous observations in other entities 30. Furthermore, the influence of pancreatic stellate cells on cancer progression in vivo is indisputable and justifies the usage of HPSC as stromal compartment in the in vitro protocol 31.

#### 3.3.2 The modified 384‐well‐device is suitable for detection of cell‐line dependent tumor‐stroma‐interactions

In a last step, the usability of our modified 384‐well‐device to detect a possible differential influence of HPSC on sphere‐formation of different PDAC was analyzed by the introduction of two doxycyclin‐inducible mutants from PaTu‐8988t and Suit2‐007 wildtype cells as a second PDAC cell line to the protocol (Fig. 3B). In comparison to the respective wildtype cells, *clone I* showed a tighter and *clone II* a looser packed shape. The possibility of cell‐line‐dependent characteristics of 3D cultures was discussed previously 23. Nevertheless, in both mutants in accordance to the wildtype cells, 60% HPSC led to the compaction of the cells to a tight spheroid. Other conditions were observed with the Suit2‐007 wildtype cells: an amount of 80% HPSC was necessary for the stromal‐induced spheroid‐formation. A differential influence of stromal cells in dependency of the cell line could be observed earlier 30.

To sum up, the modified 384‐well‐device is useable for the screening of different cell lines regarding their spheroid‐formation in the presence of stromal cells under different cultivation methods. The spheroid culture is an approved model to study tumor biology in vitro 32, and the correlation between a compact spheroid‐formation and tumorigenicity in mouse models is already confirmed 33. So the identification of a stromal‐induced spheroid‐formation can give a hint to in vivo‐like tumor‐stroma‐interactions. Findings from the screenings with the modified 384‐well‐device can act as basis for further investigations. Molecular mechanisms of the interaction can be assessed with the device e.g. by implementing feeding of supplements or potential inhibitors of the interaction.

## 4 Concluding remarks

A modified 384‐well‐device for the investigation of chemoresistance and tumor‐stroma‐interaction as key players for tumor progression was established. Compared to other commercially available devices it provides many advantages (Fig. 1). Due to the possibility of automation, a plethora of different genes can be assessed regarding their influence on tumor progression in a high‐throughput manner and with a high statistic assurance. An extension of the protocols e.g. to transcriptomic or metabolomic profiling of interesting candidates is needed to identify molecular mechanisms but nevertheless, the results from the presented protocols serve as basis for this. Requirements for the availability of the protocols in personalized medicine are also given. As a logical implication to dose‐response‐relations of defined cocultures and due to miniaturization, low‐sized samples like biopsies can be analyzed to improve patient's outcome.

**Practical application**

With the present study, a modified 384‐well‐device for investigations of tumor biology was established. The device shows versatilities regarding (i) cultivation methods, (ii) screening approaches, in detail chemo‐response and tumor‐stroma‐interaction, and (iii) operation mode, thus experimental flexibility. Besides the practicability for 2D cultures, the device was adapted to 3D cell cultivation which better mimics in vivo situations. By applying cells of different genetic background to the protocols, different genes can be analyzed regarding their influence on tumor biology. The possibility to adapt all steps to automation ensures high‐throughput analyses with a high degree of reproducibility. By introducing patient samples to the protocols, their use in therapy‐associated diagnostics could improve patient's outcome in the future.

*The authors have declared no conflict of interest*.

*The manuscript doesn't contain experiments using animals or human studies*.

## Supporting information

Supporting Information
