# Supplementary material for: A modified 384‐well‐device for versatile use in 3D cancer cell (co‐)cultivation and screening for investigations of tumor biology in vitro
Source: Eng Life Sci. 2017 Nov 24;18(2):132–9. doi: 10.1002/elsc.201700008 (PMC5873453; doi:10.1002/elsc.201700008)
Supplement: Supplementary file 1 — Supporting Information [file ELSC-18-132-s001.pdf]

Supplemental Data

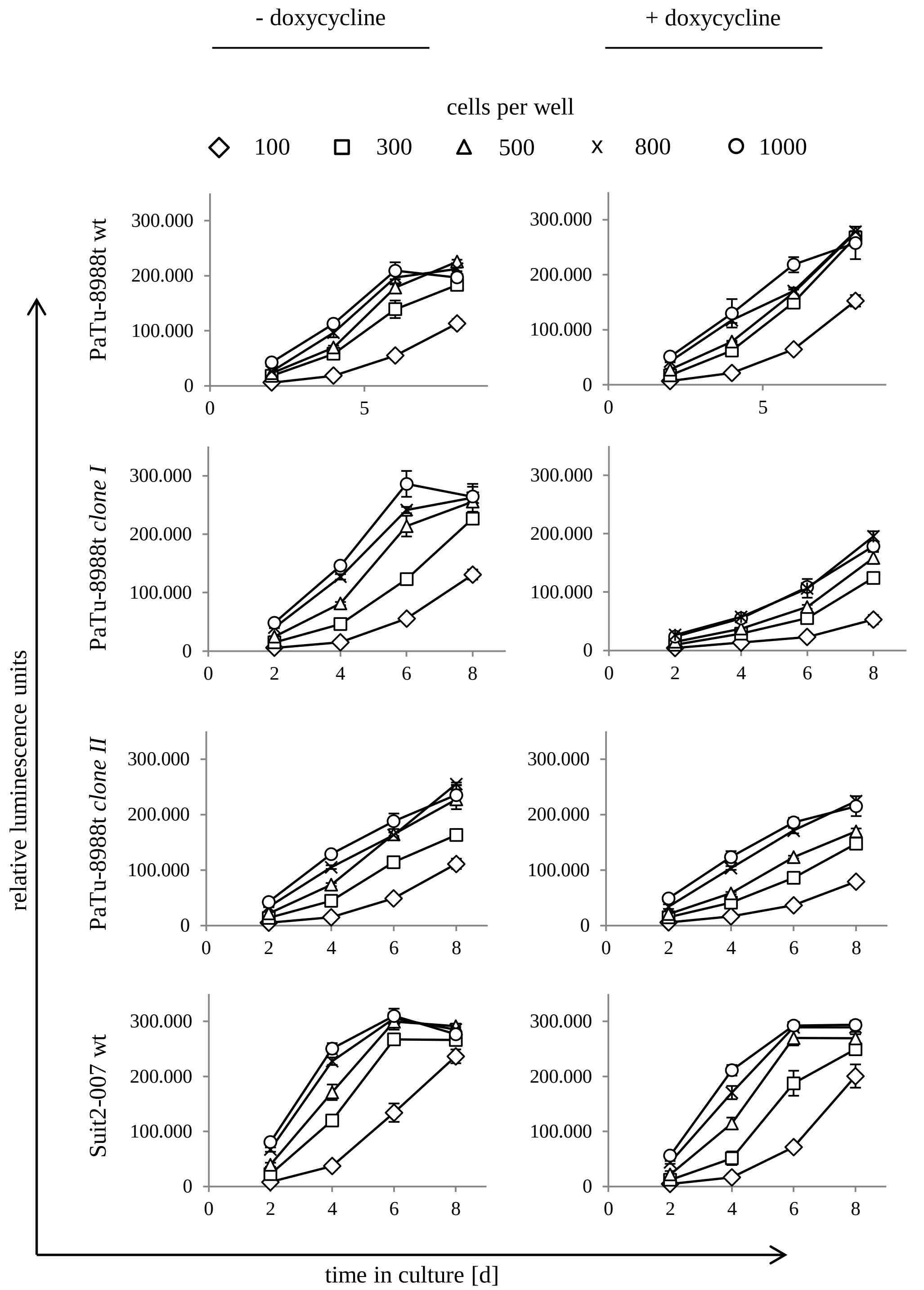

Fig. S1: Growth kinetics of different PDAC cell lines w/wo doxycycline using the LO-method. Different cell counts (◇ - 100, □ - 300, △ - 500, × - 800, ○ - 1000) were seeded per well and CellTiterGlo® 3D assays were performed on day 2, 4, 6 and 8 after seeding. Relative luminescence intensities were plotted against time. Data are depicted as mean +/- SD from one experiment with 4 samples per cell count and time point.

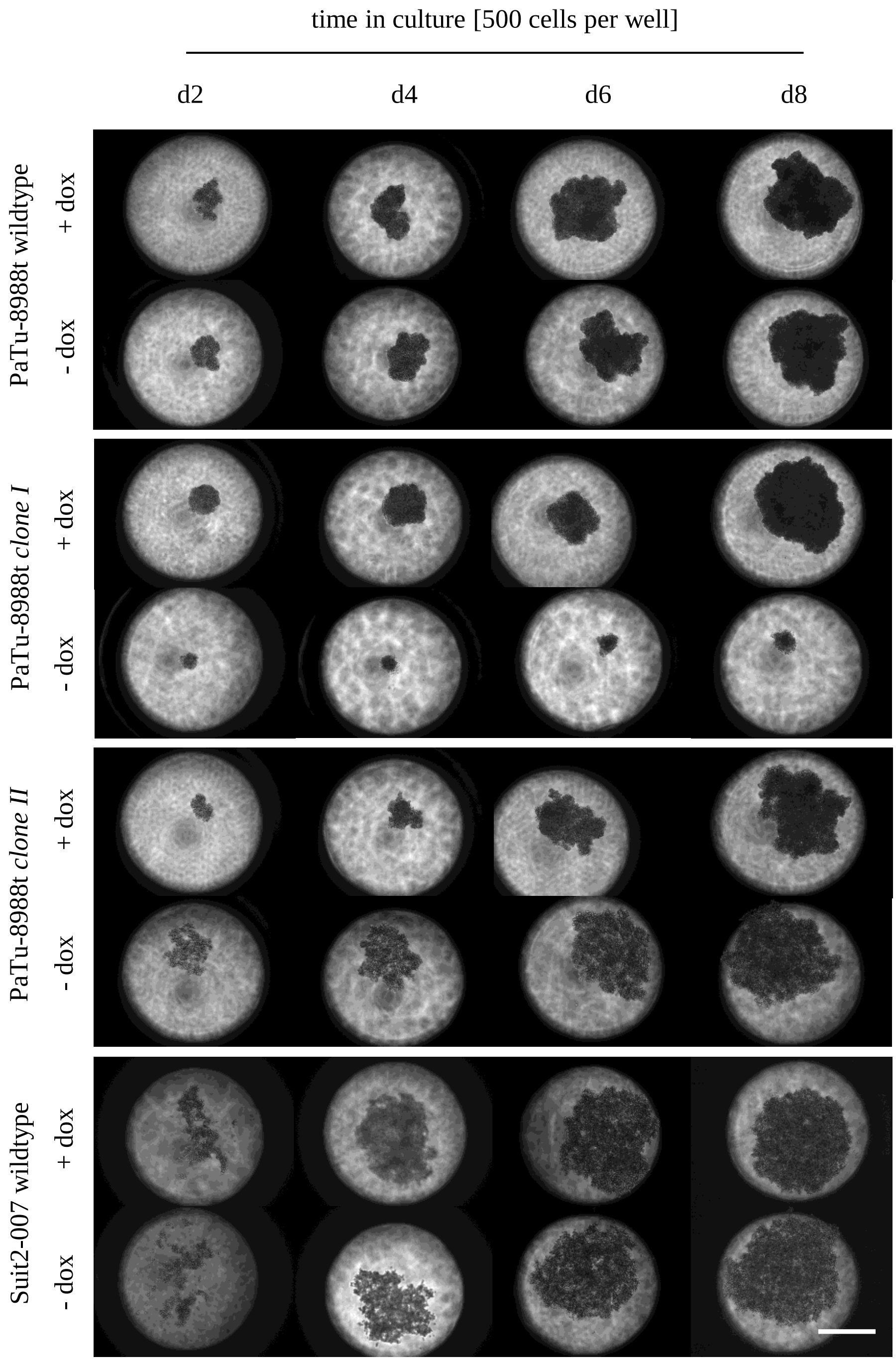

Fig. S2: Automated microscopic determination of growth kinetics of different PDAC cell lines in a density of 500 cells per well w/wo doxycycline on day 2, 4, 6 and 8 after seeding using the LO-method. Bar – 500 µm.
